# Targeting B7-H3 Immune Checkpoint With Chimeric Antigen Receptor-Engineered Natural Killer Cells Exhibits Potent Cytotoxicity Against Non-Small Cell Lung Cancer

**DOI:** 10.3389/fphar.2020.01089

**Published:** 2020-07-30

**Authors:** Shuo Yang, Bihui Cao, Guangyu Zhou, Lipeng Zhu, Lu Wang, Li Zhang, Hang Fai Kwok, Zhenfeng Zhang, Qi Zhao

**Affiliations:** ^1^ Cancer Centre, Faculty of Health Sciences, University of Macau, Macau, China; ^2^ Institute of Translational Medicine, Faculty of Health Sciences, University of Macau, Macau, China; ^3^ Department of Radiology, Translational Medicine Center and Guangdong Provincial Education Department Key Laboratory of Nano-Immunoregulation Tumor Microenviroment, The Second Affiliated Hospital of Guangzhou Medical University, Guangzhou, China; ^4^ Department of Gynecologic Oncology and Reproductive Medicine, The University of Texas MD Anderson Cancer Center, Houston, TX, United States

**Keywords:** B7-H3, chimeric antigen receptor, NK-92, immune checkpoint, natural killer cell

## Abstract

Chimeric antigen receptor (CAR)-modified natural killer (NK) cell therapy represents a kind of promising anti-cancer treatment because CAR renders NK cells activation and recognition specificity toward tumor cells. An immune checkpoint molecule, B7-H3, plays an inhibitory role in modulation of NK cells. To enhance NK cell functions, we generated NK-92MI cells carrying anti-B7-H3 CAR by lentiviral transduction. The expression of anti-B7-H3 CAR significantly enhanced the cytotoxicity of NK-92MI cells against B7-H3-positive tumor cells. In accordance with enhanced cytotoxicity, the secretions of perforin/granzyme B and expression of CD107a were highly elevated in anti-B7-H3 CAR-NK-92MI cells. Moreover, compared to unmodified NK-92MI cells, anti-B7-H3 CAR-NK-92MI cells effectively limited tumor growth in mouse xenografts of non-small cell lung cancer and significantly prolonged the survival days of mice. This study provides the rationale and feasibility of B7-H3-specific CAR-NK cells for application in adoptive cancer immunotherapy.

## Introduction

Non-small cell lung cancer (NSCLC) accounts for about 85% of all lung cancers (Oser et al., 2015). Depending on the stages of the cancer, NSCLC patients receive different treatments of surgery, radiation, chemotherapy, targeted therapies, and immunotherapy accordingly. Cancer cells frequently metastasize to some major organs, such as bone, brain, lung, and liver. Metastatic spread of cancer is the reason for most NSCLC deaths (Riihimaki et al., 2014).

The role of programmed cell death protein 1 (PD-1) as an immune checkpoint has been well elucidated in cancer therapy. At present, two anti-PD-1 antibodies (nivolumab and pembrolizumab) have been approved for NSCLC therapy (Forde et al., 2018). B7-H3 is a newly found immune checkpoint molecule (Picarda et al., 2016; Castellanos et al., 2017). Less is known about the receptor of the B7-H3 molecule. Many studies demonstrate that B7-H3 regulates the function of T cells and natural killer (NK) cells, leading to immune evasion of tumor cells (Hofmeyer et al., 2008). Beyond its inhibitory role, recent studies demonstrate that it is also frequently expressed by tumor vasculature (Kraan et al., 2014; Seaman et al., 2017). It is overexpressed in multiple tumor types, including human lung, breast, and colon cancer (Flem-Karlsen et al., 2019). B7-H3 expression is associated with tumor infiltrating lymphocytes and tumor metastasis in major cancers (Sun et al., 2006; Brunner et al., 2012). B7-H3 is an ideal target for cancer immunotherapy (Yang et al., 2020). Several anti-B7-H3 monoclonal antibodies have been studied in preclinical or clinical trials. The anti-B7-H3 antibody m276 conjugated with small molecule drugs (ADCs) has shown effective for the selective destruction of tumor vasculature in multiple tumor xenografted models (Seaman et al., 2017). Another anti-B7-H3 antibody 8H9 radiolabeled with iodine-131 has improved survival of patients with metastatic central nervous system neuroblastoma (Kramer et al., 2010). Anti-B7-H3 8H9 ADCs further showed the ability of treating NSCLC both *in vitro* and *in vivo* (Zhu et al., 2019). Recently, chimeric antigen receptor (CAR)-modified lymphocytes represent the new therapeutic forms that use artificial receptors, CARs, to redirect lymphocytes against tumor cells (Liu et al., 2019; Ma et al., 2019). Anti-B7-H3 CAR T cell therapy exhibits potent efficacy in preclinical models of tumors, including pediatric tumors, glioblastoma, melanoma, and hematologic malignancies (Du et al., 2019; Majzner et al., 2019; Nehama et al., 2019; Tang et al., 2019; Zhang et al., 2020).

NK cells are critical for innate immunity in preventing tumor metastases, which are associated with the escape from immunosurveillance (Waldhauer and Steinle, 2008). Adoptive transfer of allogeneic NK cells has been used to treat cancer in clinic for the low risk of graft-versus-host-disease (GVHD), which often occurs in the cases of allogeneic T cells (Lorenzo-Herrero et al., 2018). A human NK cell line, NK-92, was derived from patients with malignant non-Hodgkin's lymphoma (Gong et al., 1994). NK-92MI is a derivative cell line of NK-92 with transfection of human interleukin (IL)-2 (Tam et al., 1999). Unlike primary NK cells, which have the variations of expansion capability among different donors, NK-92 and NK-92MI cell lines can be continuously expanded with the similar phenotypical and functional characteristics of primary NK cells. Importantly, lack of most of the inhibitory killer immunoglobulin-like receptors (KIRs) enables NK-92 and NK-92MI cells high cytotoxicity against malignancies (Klingemann et al., 2016). Safety and antitumor activity of infused NK-92 cells have been demonstrated in preclinical models and clinical trials (Klingemann et al., 2016). A number of CAR-modified NK-92 or NK-92MI cells have been constructed toward a panel of tumor-associated antigens, including ErbB2, CD4, CD19, CD20, CD33, CD38, CD138, GD2, and epithelial cell adhesion molecule (EPCAM) (Zhang et al., 2017). These NK constructs have been demonstrated as effective treatments in preclinical models.

In this study, to enhance the potency of NK cells, we modified NK-92MI cells with an anti-B7-H3 CAR that consists of a single chain variable fragment (scFv) of the anti-B7-H3 antibody 8H9, the intracellular 4-1BB domain, and CD3ζ chain. Compared to unmodified NK-92MI cells, the activity and cytotoxicity of CAR-modified NK-92MI cells were significantly enhanced *in vitro* and *in vivo*. Our results demonstrate that redirection of anti-B7-H3 CAR promotes activation and antitumor cytotoxicity of NK cells.

## Materials and Method

### Cell Line and Cell Culture

The human lung cancer cell lines, A549, NCI-H23, HCC827, and Burkitt's lymphoma cell line Daudi, were cultured in RPMI 1640 (Cellgro). The human embryonic kidney (HEK) cell line, 293T, and the breast cancer cell line, MDA-MB-231, were cultured in DMEM (Cellgro). The human colorectal cancer cell lines, DLD-1 and HCT-116, were maintained in DMEM/F-12 medium (GIBCO). All above cell culture media were supplemented with 10% fetal bovine serum (FBS) (GIBCO), 100 U/ml penicillin, and 100 µg/ml streptomycin (GIBCO). The human NK-92MI cells were grown in alpha minimum essential medium (GIBCO) supplemented with 0.2 mM inositol (Sigma-Aldrich), 0.1 mM 2-mercaptoethanol (Sigma-Aldrich), 0.02 mM folic acid (Sigma-Aldrich), adjusting to a final concentration of 12.5% horse serum (GIBCO), 12.5% FBS, 100 U/ml penicillin, and 100 µg/ml streptomycin. All the above cell lines were maintained in conditions with 5% CO_2_ at 37°C. HEK293T, Daudi, NK-92MI, MDA-MB-231, NCI-H23, HCC827, and A549 cell lines were kindly provided by Stem Cell Bank, Chinese Academy of Sciences. DLD-1 and HCT-116 cell lines were provided by Prof. H.F. Kwok in the University of Macau.

### Purification and Expression of Anti-B7-H3 IgG 8H9

The anti-B7-H3 IgG 8H9 was expressed and purified according to the instruction as previously described (Li D. Z. et al., 2018). Briefly, the IgG 8H9 was expressed by transient transfection of the plasmids with polythyleneimine (MW25000, Polyscience) in Free-style 293-F (Invitrogen) cells. The expressed IgG proteins were purified by protein A agarose beads (GE Healthcare).

### Flow Cytometric Analysis of B7-H3 Expression

To detect B7-H3 expression on the surface of target cell lines, the cancer cell lines A549, NCI-H23, HCC827, DLD-1, HCT-116, MDA-MB-231, and Daudi were stained with anti-B7-H3 IgG 8H9 in PBS supplemented with 3% FBS for 30 min at 4°C, respectively. Then cells were rinsed and incubated with PE-conjugated goat anti-human IgG secondary antibody (Thermo Fisher Scientific) for 30 min at 4°C. After washing, cells were analyzed using the Accuri™ C6 Flow Cytometer (BD Biosciences). Flow cytometry data were analyzed with FlowJo software.

### Immunoprecipitation Assay

The cancer cell lines, A549, NCI-H23, and Daudi, were collected when grown to ~80% confluence. The lysis buffer [50 mmol/L Tris-HCl (pH 7.2), 167 mmol/L NaCl, 10% glycerol, 1% Triton X-100, and protease inhibitor cocktail tablets] was used to lyse the collected cells by incubation on ice for 20 min. The lysates were then centrifuged at 14,000 rpm for 20 min at 4°C. For immunoprecipitations, lysates were incubated with anti-B7-H3 IgG 8H9 or control IgG (5 μg/ml) overnight at 4°C. Protein A agarose beads were then added and incubated for 4 h at 4°C. Then, the beads were washed with lysis buffer and boiled with the loading buffer. The supernatants were separated in the SDS-PAGE gel, and transferred onto the PVDF membrane (Bio-Rad). After incubation with PBST containing 5% non-fat milk for 1 h at room temperature (RT), the membrane was probed with the goat anti-human B7-H3 primary antibody MAB1027 (R&D Systems) overnight at 4°C. The membrane was then incubated with the peroxidase-conjugated rabbit anti-goat IgG (H+L) (Jackson ImmunoResearch) for 1 h at RT and visualized using Clarity™ Western ECL Substrate (Bio-Rad).

### Immunohistochemistry

All procedures were in accordance with the ethical standards approved by the Guangzhou Medical University (2019-ks-23). The human lung cancer and normal tissue samples were fixed in 10% formalin and processed for paraffin embedding. The sectioned slices were deparaffined in xylene and rehydrated in graded alcohol, and placed in Tris-buffered saline (TBS) for 15 min. After antigen retrieval and inactivation of endogenous peroxidase, the sections were blocked with animal non-immune serum (Maxvision) and incubated with the goat anti-human B7-H3 primary antibody MAB1027 or 8H9 overnight at 4°C, respectively. After incubated with the corresponding secondary antibodies (Maxvision) for 15 min, the sections were stained by using the detection kit (Maxvision). Cell nucleus was stained with hematoxylin (Sigma). Finally, all sections were dehydrated with absolute ethanol. The pictures of samples were captured by the Olympus TH4-200 microscope.

### Generation of Anti-B7-H3 CAR-Expressing NK-92MI Cells

The anti-B7-H3 CAR is comprised of an immunoglobulin heavy chain signal peptide (SP), an anti-B7-H3 scFv derived from 8H9 antibody, followed by the CD8 transmembrane (TM) region, the intracellular domains of 4-1BB and CD3ζ. The codon-optimized genes were synthesized by BGI and ligated into the lentiviral expression vector, pLVX-EF1-IRES-ZsGreen, to generate a pLVX-8H9-BBζ construct. To produce the lentiviral CAR particles, 95% confluent HEK293T cells were transfected with the pLVX-8H9-BBζ plasmid together with the packaging plasmids, psPAX2 and pMD2.G. The lentiviral supernatants were collected at 72 h post the transfection and filtrated through a 0.45 µm filter (Millipore), followed by centrifugation for 2 h at 28,000 rpm. For titer calculation, HEK293T cells (5×10^5^ per well) were seeded into 12-well plate and cultured to reach 80% confluence. The different dilutions of lentiviruses were mixed with polybrene at 5 µg/ml in 1 ml fresh medium. The culture medium in the wells were replaced by the mixture of lentiviruses and incubated for 2 d. After harvested, the infection percentages of HEK293T cells were counted based on Zsgreeen detection by the Accuri™ C6 Flow Cytometer. FlowJo was used to analyze the data. The positive ratio of each well was recorded. The titer can be calculated from cell counting using the following formula:

(1) $$titer(TU/ml)=\frac{\text{Total}\;\text{cell}\;\text{number}\;\times \text{positive}\;\text{rate}}{\text{added}\;\text{Volume}\;\text{of}\;\text{lentivirus}(\mu l)}\times 1,000.$$

NK-92MI cells were adjusted to 1×10^5^ cells/ml and transduced with the concentrated lentiviral particles at a multiplicity of infection (MOI) of ~5 in the presence of 8 µg/ml polybrene, followed by incubation at 37°C and 5% CO_2_. Transduced cells were expanded and the ZsGreen-positive CAR-expressing NK-92MI cells were further enriched by ﬂow cytometric cell sorting with a FACSAria II cell sorter (BD Biosciences).

### Quantitative Real-Time PCR

Quantitative real-time PCR (qPCR) was performed to evaluate the mRNA expression of B7-H3-specific CAR in the CAR-NK-92MI cells. Total RNA samples were collected using TRIzol reagent (Thermo Fisher Scientific) from CAR-NK-92MI and NK-92MI cells, respectively. Samples were then reverse-transcribed into cDNA with iScript™ cDNA Synthesis Kit (Bio-Rad). GAPDH was used as the internal reference. The PCR was performed with 1 cycle at 94°C for 1 min followed by 45 cycles at 95°C for 30 s, at 59°C for 30 s, and at 72°C for 30 s, in total volumes of 20 μl with all reactions run in duplicate. The primer pairs were B7-H3-specific CAR-F: 5'-GGAAGCACCCAGTACAACGA-3', B7-H3-specific CAR-R: 5'-CCCCAATAGGCGAACCAAGT-3'; GAPDH-F: 5'-TGACTTCA ACAGCGACACCCA-3', GAPDH-R:5'-CACCCTGTTGCTGTAGCCA A A-3'.

### Cytotoxicity Assay

Cytotoxicity of CAR-NK-92MI cells toward various target cell lines at the different eﬀector to target (E/T) ratios was evaluated using the Calcein-AM efflux assay as described previously with some modifications (Neri et al., 2001). Briefly, A549, HCC827, NCI-H23, DLD-1, HCT-116, MDA-MB-231, and Daudi cells (5,000 cells per well in a 96-well plate) were used as target cells which were labeled with 10 µmol/L Calcein-AM (Invitrogen) for 30 min at 37°C, followed by cocultured with effector cells of amount at different E/T ratios of 10:1, 5:1, and 1:1 in total volume of 200 µl for 2 h at 37°C, respectively. Target tumor cells in the absence of ZsGreen-positive CAR-expressing NK-92MI cells were used for detection of spontaneous release. Target cells added with lysis buffer were used for detection of max lysis. After 2 h, cells were centrifuged and supernatants of cell mixture were harvested for detection. The reading values of fluorescence intensity (FI) in the supernatants were measured at excitation 495 nm and emission 515 nm using a PerkinElmer Victor X3 Microplate Reader (Perkin Elmer). The specific lysis ratios were calculated by the formula:

(2) $$lysis\;ratio\;(\%)=\frac{\text{FI}\;(\text{sample}\;\text{wells})\;-\text{FI}\;(\text{spontaneous}\;\text{release})}{\text{FI}\;(\text{max}\;\text{release})-\text{FI}\;(\text{spontaneous}\;\text{release})}\times 100\%.$$

### Degranulation and Granule Secretion Analysis

To analyze degranulation of effector cells, the cell-surface expression of CD107a and the secretion of cytotoxic granules by effector cells were analyzed by the Accuri™ C6 Flow Cytometer. Briefly, CAR-NK-92MI or NK-92MI cells were cocultured with target cells at an E/T ratio of 1:1. Monensin (Sigma-Aldrich) was added 4 h prior to 24 h with a final concentration of 2 μmol/L. After 24 h, cells were harvested and washed with PBS supplemented with 3% FBS, followed by labeled with PE-conjugated anti-CD107a antibody (Biolegend). Meanwhile, after the effector cells were cocultured with the target cells at an E/T ratio of 1:1 for 24 h, perforin and granzyme B were determined by fixed/permeabilized with Cytofix/Cytoperm solution (Beyotime), followed by stained with PE-conjugated anti-human perforin (Biolegend) and granzyme B (Biolegend) antibodies for 30 min at 4°C, respectively. The cells were analyzed by flow cytometry and all data were analyzed with FlowJo software.

### *In Vivo* Tumor Growth Studies

All animal experiments were in accordance with the ethical standards approved by the University of Macau (UMARE-018-2017). NOD/SCID mice (6–7 weeks old) were provided by the animal research core of University of Macau. The A549 xenografts were established by injecting subcutaneously at the right flank of mice with 210^6^ cells. Tumor cell engraftments were monitored by caliper measurements. At 10 d post subcutaneous inoculation, tumor-bearing mice received treatments with 5×10^6^ of CAR-NK-92MI cells, unmodified NK-92MI cells, and PBS weekly for 4 weeks, respectively. The tumor volumes were measured and calculated according to the formula:

(3) $$tumor\;volume=\frac{{\text{width}}^{2}\times \text{length}}{2}.$$

### Statistical Analysis

Statistics was analyzed using the GraphPad Prism software. Data are obtained from duplicate or triplicate samples in 2–3 individual experiments. Significant differences were calculated by two ways ANOVA, t tests, nonparametric Mann-Whitney test, or log-rank test. The values of * *p* < 0.05, ** *p* < 0.01, and *** *p* < 0.001 were set as the standard for statistical significance levels.

## Results

### Expression of B7-H3 in Human Cancer Tissues and Cell Lines

**Figure 1:**
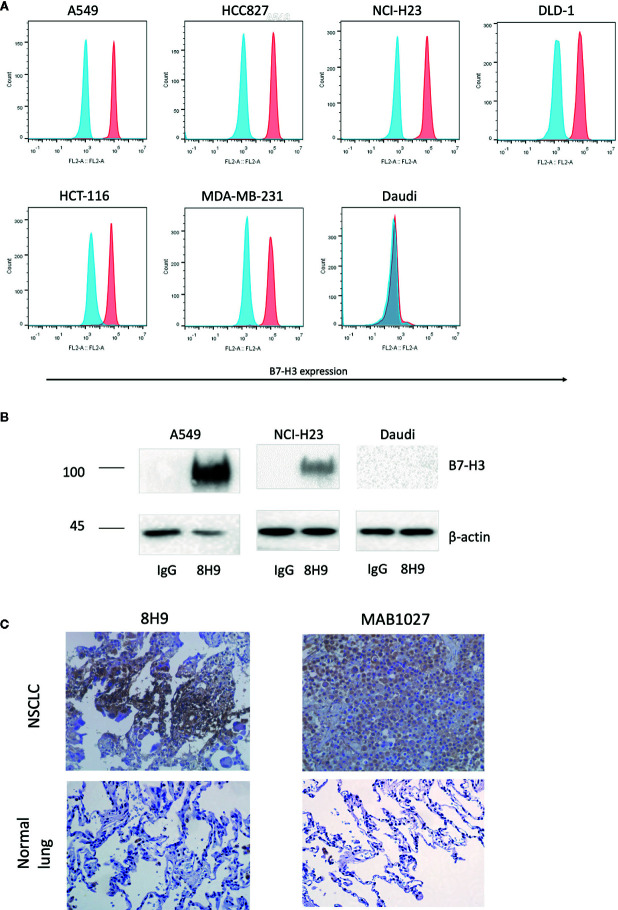
Expression of B7-H3 on tumor cell lines and primary human tissues. **(A)** Flow cytometric analysis of B7-H3 expression on the surface of different target cell lines was detected with the anti-B7-H3 8H9 IgG. Red color represents 8H9 IgG staining. Blue color represents control IgG staining. **(B)** Immunoprecipitation of B7-H3 in A549, NCI-H23, and Daudi cell lines. The cell lysates were immunoprecipitated by the 8H9 IgG or control IgG, followed by western blot using the anti-B7-H3 antibody, MAB1027. β-actin levels were utilized as the loading control. **(C)** Immunohistochemistry (IHC) of human NSCLC tissues and normal lung tissues. Tumor tissue sections were stained with the anti-B7-H3 8H9 IgG or the anti-B7-H3 antibody MAB1027. Images were taken under ×400 magnification.

We firstly assessed the expression of B7-H3 in different cancer cell lines with the anti-B7-H3 IgG 8H9 using flow cytometric analysis and immunoprecipitation assay. Flow cytometric analyses (**Figure 1A**) demonstrated that B7-H3 was highly expressed on the cell surface of several cancer cell lines, A549, NCI-H23, HCC827, DLD-1, HCT-116, and MDA-MB-231, except the B7-H3-negative cell line (Daudi). Western blot analysis (**Figure 1B**) further confirmed that the 4Ig-B7-H3 protein with ~100 kDa was immunoprecipitated from whole cell lysates of A549 and NCI-H23 but not Daudi by the 8H9 antibody. As shown in **Figure 1C**, immunohistochemistry results showed that both the 8H9 antibody and the commercial anti-B7-H3 antibody (MAB1027) detected B7-H3 in the human NSCLC tissues. No positive staining was detected in the normal lung tissues. Above data suggest that B7-H3 is highly expressed in human solid tumor cell lines and tissues. The 8H9 antibody exhibited the strong reactivity toward B7-H3 without cross-reactivity to normal lung tissues. The antibody 8H9 was thus chosen for the CAR construction.

### Generation of NK-92MI Cells Carrying the Anti-B7-H3 CAR

**Figure 2:**
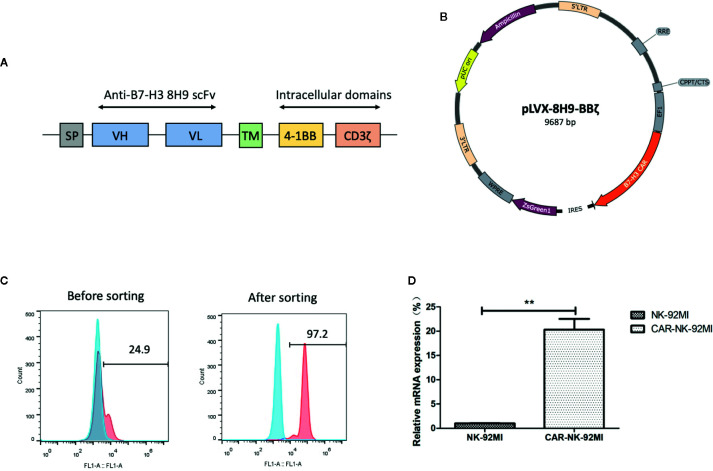
Generation of B7-H3-specific CAR-NK-92MI cells. **(A)** Schematic representation of the lentiviral vector loaded with B7-H3-specific CAR. The B7-H3-specific CAR encodes an immunoglobulin heavy chain signal peptide (SP), the anti-B7-H3 scFv derived from 8H9 antibody, followed by the CD8 transmembrane (TM) region, the 4-1BB domain, and the CD3ζ chain. **(B)** Diagram of the B7-H3-CAR lentiviral vector containing the major functional elements. **(C)** Flow cytometric analysis of CAR expression on the surface of transduced NK-92MI cells before and after cell sorting. CAR-NK-92MI cells were sorted by GFP with unmodified NK-92MI cells set as the control. **(D)** Expression of the B7-H3 CAR in NK-92MI cells by qPCR analysis. Primers anchoring in the region of 8H9 scFv were designed to measure relative mRNA expression level in CAR-NK-92MI cells compared to NK-92MI cells. **p < 0.01.

The anti-B7-H3 scFv was generated by linking heavy and light chain variable domains of the anti-B7-H3 antibody 8H9 *via* a (G4S)_3_ linker as previously described (Ahmed et al., 2015). The anti-B7-H3 CAR construct was designed to contain the anti-B7-H3 scFv, the CD8 TM region, the 4-1BB, and CD3ζ intracellular domains (**Figure 2A**). The CAR DNA sequence was inserted into the pLVX lentiviral vector with the co-expression of ZsGreen (**Figure 2B**). The lentiviral particles were subsequently produced and used for transduction of human NK-92MI cells. As shown in **Figure 2C**, transduction efficiency was 24.9%. After several rounds of cell sorting, the percentage of CAR-expressing NK-92MI cells exceeded 95%. Expression of the anti-B7-H3-CAR was examined by qPCR. As shown in **Figure 2D**, the CAR was highly expressed in the transduced CAR-NK-92MI cells compared to untransducted NK-92MI cells.

### Enhanced Cytotoxicity of CAR-NK-92MI Cells Against B7-H3-Positive Tumor Cell Lines

**Figure 3:**
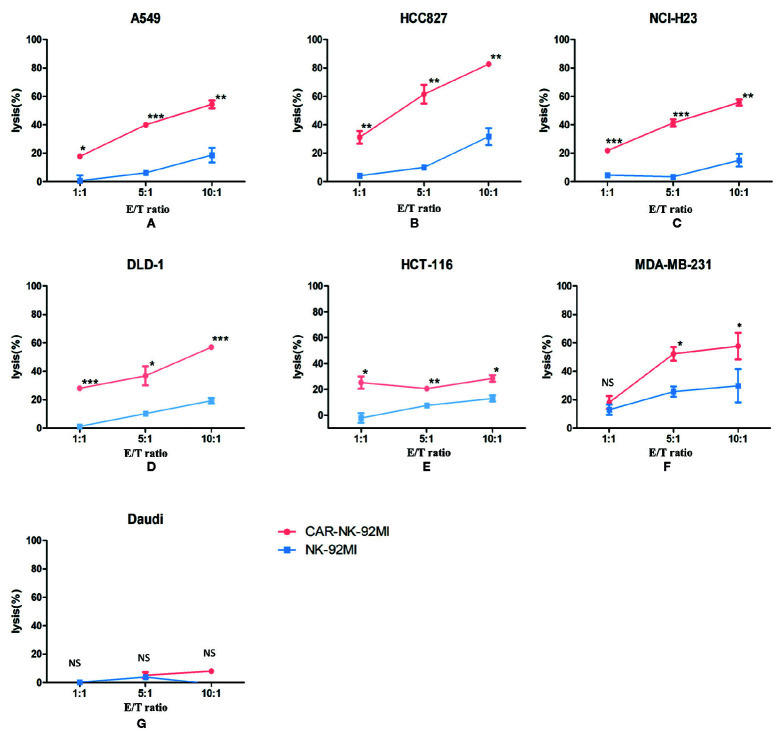
Cytotoxicity assay of B7-H3 CAR-NK-92MI cells toward a panel of tumor cell lines *in vitro*. Significant killing was observed in B7-H3-positive cell lines A549, HCC827, NCI-H23, DLD-1, HCT-116, and MDA-MB-231 but not in B7-H3-negative cell line Daudi. The effector cells were co-cultured with target cells for 2 h at different E/T ratios as indicated. Data represent one representative experiment from three individual experiments. ** p < 0.001; **p < 0.01; *p < 0.05.

To determine whether B7-H3 recognition could improve the cytotoxicity of NK-92MI cells, we compared CAR-NK-92MI cells and unmodified NK-92MI cells in response to a panel of human cancer cell lines by a Calcein-AM assay. As shown in **Figure 3**, the cytotoxicity abilities of CAR-NK-92MI cells was significantly enhanced at all E/T ratios compared with unmodified NK-92MI cells. However, toward MDA-MB-231, there was no significant difference between the effectiveness of CAR-NK-92MI and unmodified NK-92MI cells at low E/T ratio of 1: 1. B7-H3-negative Daudi cells were not lysed by any of the NK-92 derivatives. When these cell lines were examined, unmodified NK-92MI cells had moderate killing against HCC827 and MDA-MB-231 cell lines at high E/T ratio, which are however lysed by CAR-NK-92MI cells with statistically significant differences. These cell lines are intrinsically sensitive to NK cells, which is consistent with other reports (Dominguez et al., 2016; Shenouda et al., 2017). Taken together, these results_ indicated that the B7-H3 CAR-NK-92MI was cytotoxic specifically against B7-H3-positive tumor cells.

### Elevated Activation of CAR-NK-92MI Cells in Response to B7-H3-Positive Target Cells

**Figure 4:**
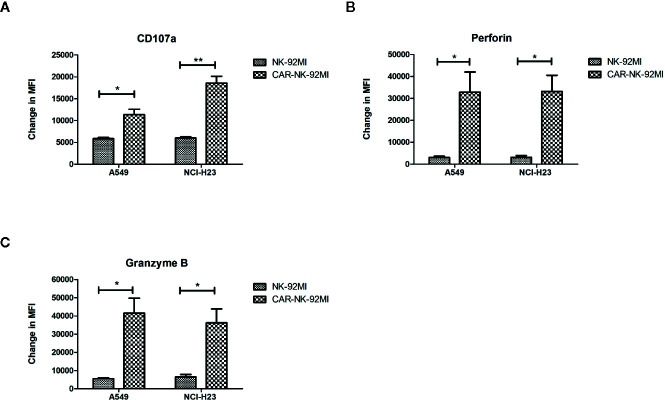
Degranulation and granule secretion of B7-H3 CAR-NK-92MI cells. **(A)** Surface expression of CD107a, as measured by mean fluorescence intensity (MFI), detected in CAR-NK-92MI cells compared to NK-92MI cells in response to A549 or NCI-H23 cells. **(B, C)** Secretion of perforin and granzyme B from CAR-NK-92MI cells compared to NK-92MI cells using flow cytometric analysis. Data are presented as mean ± SEM of triplicate samples. **p < 0.01; *p < 0.05.

A hallmark of NK cell activation is degranulation in which lytic granule contents (perforin and granzymes) are released onto the surface of the target cells. CD107a, granzyme B, and perforin are three major markers in degranulation. To further analyze degranulation of CAR-NK-92MI cells, we examined the expression of CD107a and the secretion of perforin/granzyme B by flow cytometry. CAR-NK-92MI cells were incubated with A549 and NCI-H23 cells at E/T ratio of 1:1 for 24 h, respectively. The significant differences were observed in the CD107a expression on surface of CAR-NK-92MI cells compared to unmodified NK-92MI (p<0.05) (**Figure 4A**). In addition, the B7-H3 CAR strengthened expression of perforin/granzyme B in degranulation of NK-92MI cells. The levels of granzyme B and perforin were significantly elevated in CAR-NK-92MI cells in response to the target cell lines (p<0.05) (**Figures 4B, C**). Although A549 cells express much higher B7-H3 than NCI-H23 cells, the level of CD107a in A549 seems to be lower than that of NCI-H23, implying cytolytic granule polarization and degranulation are not determined by B7-H3 density above a threshold. We assume that the different receptors control degranulation in the two cell lines. Therefore, the results suggested that degranulation activation played an important role in cytotoxicity of CAR-NK-92MI cells.

### Therapeutic Efficacy of CAR-NK-92MI Cells in NSCLC Xenografts

**Figure 5:**
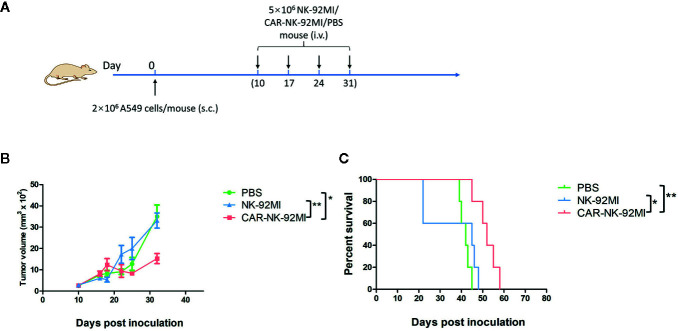
Therapy of NSCLC xenografts with B7-H3 CAR-NK-92MI cells. **(A)** Treatment procedures of NOD/SCID mice inoculated subcutaneously with A549 cells. **(B)** Tumor growth in NOD/SCID mice with treatments of CAR-NK-92MI cells compared to NK-92MI cells (**p < 0.01) and PBS (***p = 0.001). **(C)** Survival of mice by Kaplan-Meier survival analysis for CAR-NK-92MI cells compared to NK-92MI cells (*p < 0.05) and PBS (**p < 0.01). n = 5 each group.

To assess *in vivo* anti-tumor activity, the subcutaneous model of A549 cells was established. Ten days after the implantation, three groups of A549-xenografted mice received intravenous injections of CAR-NK-92MI cells (5 × 10^6^/dose), unmodified NK-92MI cells (5 × 10^6^/dose), and PBS at day 10, 17, 24, and 31, respectively (**Figure 5A**). As shown in **Figure 5B**, the group of CAR-NK-92MI reduced tumor volume by 52.1% and 63.8% at the end of the experiment compared with the NK-92MI group (p = 0.002) and the PBS group (p = 0.001), respectively. The survival time in the CAR-NK-92MI treated group was significantly longer than the PBS group (p<0.01) and the NK-92MI group (p<0.05) (**Figure 5C**). Therefore, CAR-NK-92MI cells effectively inhibited tumor growth and prolonged the survival of the tumor-bearing mice.

## Discussion

NK cells are important innate immune effectors in the control of malignancies. Their activation is regulated by the interaction with suppressive and stimulatory receptors (Kwon et al., 2017). B7-H3 plays an inhibitory role in the regulation of NK cells. Recent studies have shown that inhibition of B7-H3 augments tumor eradication by enhancing function of cytotoxic lymphocytes (Lee et al., 2017). Our previous studies demonstrated that the anti-B7-H3 antibody 8H9 inhibited B7-H3-positive tumor cells through the antibody-dependent cell-mediated cytotoxicity (ADCC) function (Ahmed et al., 2015). ADCC is a mechanism of cell-mediated immune defense through the antibody Fc region (Ochoa et al., 2017). NK cells are key components in the ADCC function of the antibody Fc (Seidel et al., 2013).

Recent studies have shown that the CAR mediates stronger NK cell response than the antibody Fc (Boissel et al., 2013). In this study, we constructed the second-generation CAR consisting of 4-1BB costimulatory signal domain and a CD3ζ domain, which is similar to the clinically used anti-CD19 CAR-T cells (Li S. et al., 2018). Our results confirmed that the anti-B7-H3 CAR directly triggered the intrinsic cytolytic function of NK cells through the signals from 4-1BB and CD3ζ chain. The *in vitro* cytotoxicity assays demonstrated that CAR-NK-92MI cells specially killed tumor cell lines depending on B7-H3 recognition. In addition, CAR-NK-92MI cells significantly enhanced cytotoxic ability against all B7-H3-positive cell lines. The redirection of anti-B7-H3 CAR further strengthened NK activation and degranulation. As an important process of NK cell activation, degranulation is triggered to release lytic granule molecules and subsequently induce apoptosis of targeted cells. The secretion of granules, such as granzyme B and perforin, is a significant indicator of immune activation for cytotoxic lymphocytes (Mellor-Heineke et al., 2013). In response to B7-H3-positive target cells, the perforin-granzyme pathway was activated. Surface CD107a is a marker of degranulation in NK cells (Cohnen et al., 2013). Besides perforin/granzyme B, we observed that CD107a expression is highly elevated in CAR-NK-92MI cells. Moreover, in NSCLC xenografts, CAR-NK-92MI cells exhibited effectively anti-tumor ability and prolonged the survival of the tumor-bearing mice.

Anti-CD19 CAR-T cell therapy shows promising therapeutic activity in hematological malignancies. However, the resistances limit the application of CAR-T therapy in solid tumor due to some mechanisms. Likewise, the solo treatment of the B7-H3 CAR incompletely prevents tumor growth in xenografts of NSCLC and colon cancer (Huang et al., 2020). Although the mechanisms have not been completely elucidated, other immune checkpoint pathways, such as PD-1/PD-L1, may be involved in the activation of anti-B7-H3 CAR-T cells. Although CAR-NK-92MI cells lack the long-term persistence, multiple administrations of CAR-NK-92MI cells can overcome this disadvantage. Because irradiated NK-92MI cells are necessary for patient infusion to ensure safety, we need to further investigate the effects of irradiation on intrinsic features of CAR-NK-92MI cells before they can be applied in clinical trials.

## Conclusion

Taken together, B7-H3 may serve as a target for NSCLC therapy. Anti-B7-H3 CAR-NK-92MI cells showed significant treatment efficacy both *in vitro* and *in vivo*. Our studies support the development of CAR-modified NK-92 as an option for adoptive cancer immunotherapy.

## Data availability statement

The datasets generated for this study are available on request to the corresponding authors.

## Ethics statement

The studies involving human participants were reviewed and approved by human ethics committee, the Guangzhou Medical University. The animal study was reviewed and approved by Animal Ethics Committee, University of Macau.

## Author contributions

SY conceived and executed experiments and co-wrote the manuscript. BC and LW conceived and executed IHC assays. GZ executed experiments related to antibody expression and purification. HK assisted cytotoxicity assays. LZhu and LZha assisted flow cytometric analysis. ZZ supervised part of the investigation. QZ supervised all aspects of the investigation and co-wrote the manuscript.

## Funding

This study was supported by the National Key R&D Program of China (2019YFA0904400), the Science and Technology Development Fund of Macau (File no. FDCT/131/2016/A3, FDCT/0015/2018/A1, FDCT/0055/2019/A1), and the intramural research program of Faculty of Health Sciences, University of Macau (File no. MYRG2019-00069-FHS).

## Conflict of Interest

The authors declare that the research was conducted in the absence of any commercial or financial relationships that could be construed as a potential conflict of interest.
